# Evaluation of quantitative polymerase chain reaction for detecting *BRCA1* or *BRCA2* copy number loss in high-grade serous ovarian cancer

**DOI:** 10.1038/s41598-025-34516-z

**Published:** 2026-01-10

**Authors:** A. J. Oswald, R. L. Hollis, M. Churchman, I. Croy, A. Ewing, J. P. Thomson, L. J. Stillie, E. Merry, M. Albertella, J. V. Forment, P. Roxburgh, C. A. Semple, C. S. Herrington, C. Gourley

**Affiliations:** 1 The Nicola Murray Centre for Ovarian Cancer Research, Cancer Research UK Scotland Centre, Institute of Genetics and Cancer, University of Edinburgh, Edinburgh, UK; 2 MRC Human Genetics Unit, Institute of Genetics and Cancer, University of Edinburgh, Edinburgh, UK; 3 Cancer Research UK Beatson Institute, Institute of Cancer Sciences, University of Glasgow, G61 1BD Glasgow, UK; 4 Bioscience, Oncology R&D, AstraZeneca, Cambridge, UK; 5 School of Cancer Sciences, Wolfson Wohl Cancer Research Centre, University of Glasgow, Glasgow, UK

**Keywords:** Copy number loss, Quantitative polymerase chain reaction, High grade serous ovarian cancer, BRCA1/2, Biomarkers, Cancer, Genetics, Molecular biology, Oncology

## Abstract

Copy number loss (CNL) of tumour suppressors genes, such as *BRCA1/2*, are frequent but under-researched tumour driver events. Accurate identification of *BRCA1/2* CNL has potential therapeutic implications, particularly in high grade serous tubo-ovarian carcinoma (HGSOC), and cost-effective detection methods are desirable. This study assessed quantitative PCR (qPCR) as a method of *BRCA1/2* CNL detection in HGSOC, in two patient cohorts (cohort 1 *n* = 355, cohort 2 *n* = 86) and a cell line panel (*n* = 10) using sequencing-based copy number (CN) assessment as a comparator (panel-based sequencing in cohort 1; whole genome sequencing [WGS] in cohort 2 and the cell line panel). The qPCR sensitivity for detecting *BRCA1/2* CNL in both clinical cohorts was poor (*BRCA1;* 0-11.5%, *BRCA2;* 10-36.8%). There was frequent co-occurrence of *BRCA1* and *BRCA2* CNL by qPCR (cohort 1; OR 71.8), which was not mirrored in matched sequencing data. There was a significantly higher CN value for the reference gene *RPPH1* in cases with qPCR-identified *BRCA1/2* co-loss (cohort 1; *RPPH1* median CN 5.2 ± 4.8 versus 2.5 ± 1.3, *p* < 0.0001). In a cell line panel, there was poor agreement between *BRCA1/2* CN measured by qPCR versus WGS, and an additional reference assay *(TERT)* did not improve agreement. Overall, qPCR did not effectively detect *BRCA1/2* CNL in HGSOC, which may be due to CN variation at the reference assay sites. This work underscores the need for caution when utilising qPCR for detecting CNL events, particularly in genomically unstable cancers.

**Supplementary Information:**

The online version contains supplementary material available at 10.1038/s41598-025-34516-z.

## Introduction

Structural variants (SVs) represent a diverse class of genomic events that may amplify, delete or rearrange genomic regions.^1^ Among SVs, large scale genomic gains and losses that generate copy number variation are common, and typically involve oncogene amplification or loss of tumour suppressor genes.^2^ Despite being less well characterised compared to short pathogenic variants (mutations), these alterations are recognised to play a critical role in tumourigenesis by affecting gene dosage and regulatory mechanisms^3,4^, with recent studies suggesting at least 30% of cancer genomes harbour pathogenic SVs.^1,5^.

Accurately identifying copy number variants in tumours has significant clinical implications, as these events can be potentially leveraged therapeutically.^1^ For example, SVs such as *NTRK* fusions and *HER2* copy number amplification have been successfully therapeutically targeted across multiple cancer types.^6,7^ Furthermore, copy number variants serve as valuable prognostic or predictive biomarkers; for example, *CCNE1* copy number gain is associated with chemoresistance in high grade serous tubo-ovarian carcinoma (HGSOC).^8,9^ Despite the growing understanding of oncogenic copy number gain events, research on copy number loss events in tumour suppressor genes has been comparatively limited.^3,10^.

Copy number loss of *BRCA1* and *BRCA2* is of great interest following the identification of frequent *BRCA1/2* SVs in HGSOC.^11–13^ The majority of these events were multi-megabase deletions spanning the gene enterity.^11^ *BRCA1/2* SV deletions occurred in 14–16% of HGSOC cases, and also at lower frequencies in other tumour types.^11,13^ Furthermore, these events were associated with reduced gene expression and a higher genomic homologous recombination deficiency (HRD) score.^11^ Further in vitro work in cell lines with *BRCA1/2* copy number loss demonstrated functional HRD and PARP inhibitor sensitivity in a range of tumour types.^14^ The accurate identification of *BRCA1* or *BRCA2* deletion SVs through copy number loss detection could therefore have high clinical utility in identifying patients most suitable for PARP inhibitors. Furthermore, *BRCA1/2* copy number loss identification may be valuable for prognostication, as *BRCA2* copy number loss is predictive of prostate cancer post-operative recurrence^15^ and *BRCA2/RB1* co-loss is associated with an aggressive prostate cancer phenotype.^16,17^.

Previous studies which identified *BRCA1/2* deletion SVs in patients with HGSOC utilised matched tumour-normal whole genome sequencing (WGS) data.^11,13^ However, routinely accessing high quality sequencing data in the clinic may pose challenges in relation to availability (particularly of fresh tissue), cost constraints, time delays, and result interpretation. Current clinically available diagnostic tests for CNL is also limited by cost and is time-consuming.^18^ Therefore, the concept of a simple, effective and cost-efficient laboratory method to detect these events is appealing. Quantitative polymerase chain reaction (qPCR) is a straight-forward, frequently utilised assay for relative quantification of nucleic acids, and has not been previously directly evaluated as a method of detecting *BRCA1/2* copy number loss. Therefore, this study assessed qPCR, using routinely available commercial assays, as a method to detect *BRCA1* or *BRCA2* copy number loss in HGSOC patients cohorts and a panel of HGSOC cell lines, against matched sequencing data.

## Materials and methods

### Cohort 1

Cohort 1 (*n* = 355) consisted of the previously molecularly characterised formalin-fixed paraffin embedded (FFPE) cohort described by Hollis et *al*.^9^ This included cases with pathologically confirmed HGSOC treated at the Edinburgh Cancer Centre. DNA extraction was performed using the QIAamp FFPE DNA Kit and Qiagen Deparaffinization Solution (Qiagen) as per manufacturer’s instructions. Extracted DNA was quantified by high sensitivity Qubit assay. Copy number data was obtained from tumour sequencing data from a custom 84-gene integrated DNA Technologies (IDT) gene capture panel (supplementary methods). The median per-sample mean target coverage was 593x. Reads were processed using bcbio v1.0.6 sequencing analysis pipeline and aligned to GRCh38. Copy number analysis was performed using CopywriteR,^19^ as outlined in supplementary methods. Transcriptomic data were also available from the previous study;^9^ briefly, RNA was extracted from macrodissected FFPE samples, cDNA was amplified, fragmented and labelled before hybridisation to the ovarian DNA sequence array cDNA microarray platform. The raw transcriptomic data underwent normalization using robust multi array average method. Gene expression analyses included only *BRCA*-wildtype patients (as *BRCA1/2* mutations have the potential to influence transcriptomic profiles).

Cellularity of analysed specimens was recorded as part of the pathological review process in the original molecular profiling study, calculated as the proportion of malignant epithelial cells on haematoxylin and eosin (H&E) FFPE macro-dissection sections. Cellularity was recorded in 20% range estimates (0–20, 21–40, 41–60, 61–80, 81–100).

### Cohort 2

Cohort 2 (*n* = 86) consisted of patients from the Scottish High Grade Serous Ovarian Cancer Whole Genome study, as previously reported (European Genome Archive S00001004410).^11,20^ Fresh frozen tumour samples underwent pathology review to confirm HGSOC diagnosis. DNA extraction and quality control were performed as previously described^11^, and samples were subsequently whole genome sequenced on the HiSeq X Ten Illumina NovaSeq 6000. CNV analysis was performed using CNVkit (version 0.9.3)^21^ and bioinformatic processing is outlined in supplementary methods. Cellularity was estimated during histopathological review and demonstrated excellent concordance with tumour cellularity measured by genomic methods (CLImAT and p53 variant allele frequency).^11^.

### Cell lines

Ten HGSOC cell lines were purchased commercially (JHOS2: Riken; SNU119: Korean Cell Line Bank) or sourced in-house at the University of Edinburgh (59 M, ES2, IGROV1, OV90, OVCAR3, OVCAR4, OVCAR8, OVSAHO). Cell lines were authenticated by short tandem repeat (STR) profiling and compared to the American Type Culture Collection (ATCC) STR database, in accordance with the National Standards Institute & ATCC standards.^22^ Cell lines with known *BRCA1* or *BRCA2* copy number loss previously identified in the literature were included as reference samples (SKES1 bone sarcoma cell line, *BRCA1* copy number loss; SKUT1 soft tissue sarcoma cell line, *BRCA2* copy number loss), based on heterozygous copy number loss identified by WGS. Cell lines were grown in the manufacturer’s suggested media with 10% foetal bovine serum without antibiotics. All cell lines were grown and maintained at 37 °C with 5% CO_2_ and passaged routinely. DNA extraction was performed from cell line pellets using the Qiagen DNeasy blood & tissue kit as per manufacturer’s instructions.

The HGSOC cell lines and reference cell lines had been previously sequenced by the Broad Institute, on Illumina HiSeq 2000, with publicly available WGS files.^23^ CNV analysis was carried out using CNVkit (version 0.9.3)^21^ and bioinformatic processing is outlined in supplementary methods.

### Quantitative PCR for copy number of BRCA1/2

Copy number of *BRCA1/2* was quantified using TaqMan genotyping commercially available qPCR copy number assays, which included two probes each targeting one end of the respective *BRCA1* and *BRCA2* gene. Probes were selected to have similar amplicon sizes and amplification efficiency was tested (> 95%). The recommended standard reference assay RNaseP reference assay *(RPPH1)* was used (pre-designed, commercially available).^24^ Despite it being common practice to have one reference assay for qPCR for copy number analysis^24^, an additional commercial pre-designed reference assay (*TERT*) was also included for the cell line cohort. This was included to explore potential issues with the standard reference assay, such as copy number variation at the reference gene loci. qPCR was conducted on a StepOne Plus Real-Time PCR System with data analysed via StepOne software (version 2.3) and CopyCaller (version 2.1), as described in supplementary methods. *BRCA1* or *BRCA2* copy number was averaged across probes, with heterozygous loss defined as absolute copy number ≤ 1.2.

### Analysis & statistics

Statistical analyses were performed using R version 4.3.2. Values are reported as median ± standard deviation for non-parametric data. Normality was assessed using the Shapiro-Wilk test. Mann-Whitney U tests were used to compare continuous variables following the identification of non-normality. Correlation analysis was conducted using Spearman’s rank correlation for non-parametric data. ANOVA was used to assess variance among multiple groups. Fisher’s exact test was used for categorical variables and odds ratio calculation. Sensitivity and specificity were calculated using the “binom” R package.

### Ethics statement and patient consent

This study was carried out in accordance with the principles of the Declaration of Helsinki. For cohort 1, ethical approval had been previously obtained from South East Scotland Human Annotated Bioresource (Lothian NRS Bioresource Ethics Committee reference 15/ES/0094-SR705 and SR752), as previously reported.^9^ The requirement for consent was waived by the ethics committee due to the retrospective nature of the study.^9^ For cohort 2, ethical approval for the use of human tissue specimens for research had also been obtained from the Lothian NHS Research Scotland Human Annotated Bioresource (ethics committee reference 15/ES/0094-SR494), as previously published.^11^ All relevant ethical regulations were complied with, including the requirement for written informed consent where required.

## Results

Two probes targeting each end of the *BRCA1* or *BRCA2* gene were used, and internal control samples (non-HGSOC cell lines and DNA from normal human tissue) confirmed the accuracy of the *BRCA1/2* assays (Supplementary Fig. 1A).

### Distribution of qPCR-detected *BRCA1*/*2* copy number loss (cohort 1)

Copy number by qPCR was assessed across a cohort of pathologically confirmed HGSOC (*n* = 355) with pre-existing gene sequencing (hybrid capture panel sequencing) and transcriptomic characterisation. There was excellent correlation between the copy number estimates from the two probes for both *BRCA1* (rho = 0.92 [95% CI 0.90–0.93]) and *BRCA2* (rho = 0.88 [95% CI 0.85-90], Supplementary Fig. 1B). The median absolute copy number measured by qPCR was slightly higher for *BRCA1* than for *BRCA2* (2.23 ± 0.88 versus 1.72 ± 0.74, Supplementary Fig. 1C). Twenty-two samples (6.4%) demonstrated *BRCA1* copy number loss and 60 samples (16.9%) demonstrated *BRCA2* copy number loss. Almost all (20/22) samples exhibiting *BRCA1* copy number loss also demonstrated concurrent *BRCA2* copy number loss. Samples with *BRCA1* copy number loss were significantly more likely to also exhibit *BRCA2* copy number loss (OR 71.8 [95% CI 16.5-653.6]).

### Comparison with genome-wide copy number estimates from sequencing data (cohort 1)

**Fig. 1:**
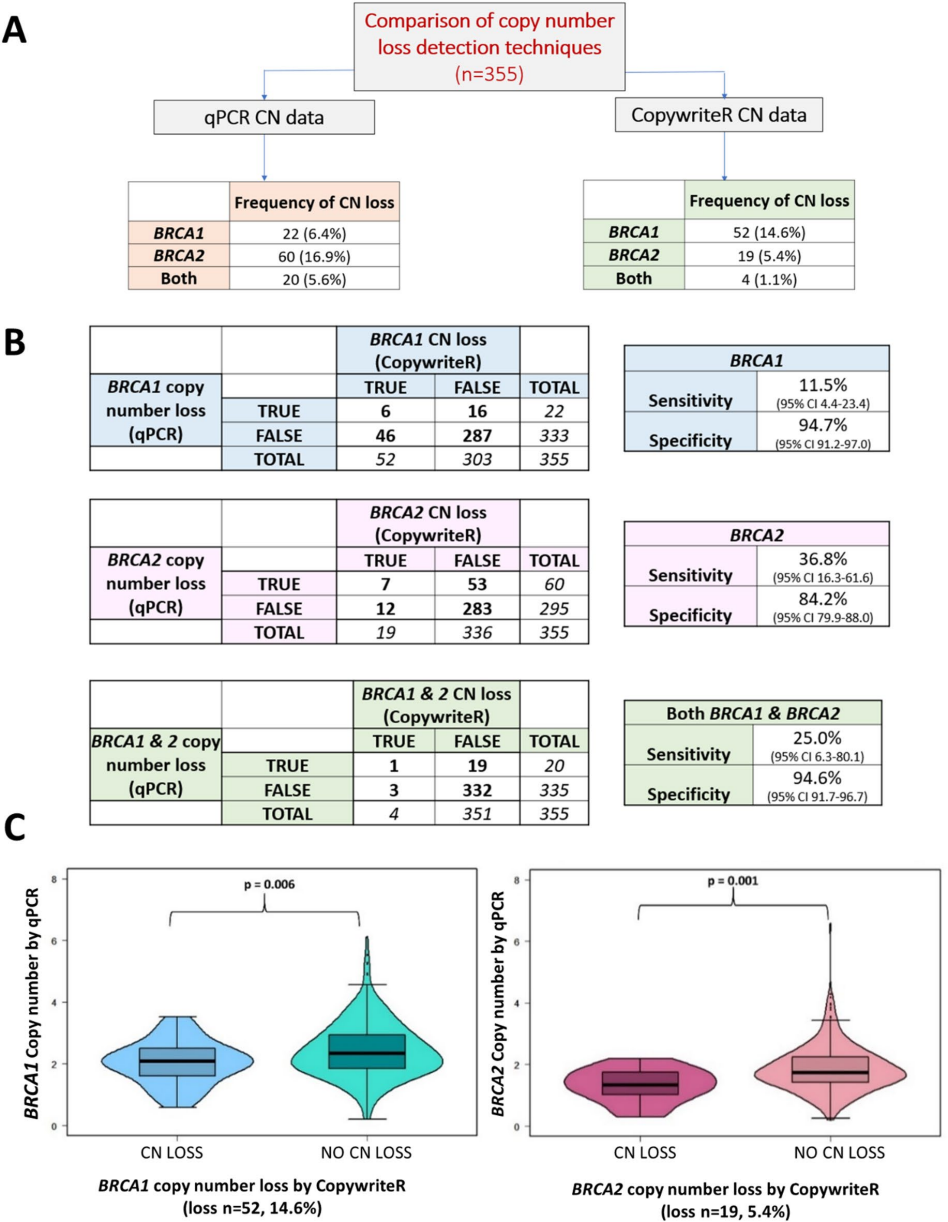
**(A)** Frequency of copy number (CN) loss events of *BRCA1* and *BRCA2* based on the qPCR and CopywriteR method in cohort 1 (*n* = 355). **(B)** The sensitivity and specificity of qPCR as a method for detecting copy number loss, using CopywriteR method CopywriteR as a benchmark in cohort 1 (*n* = 355). **(C)** Copy number value by qPCR, when samples were grouped as *BRCA1* or *BRCA2* copy number loss by CopywriteR.

To assess the sensitivity of qPCR as a method for detecting copy number loss, *BRCA1/2* copy number was measured using genome-wide copy number estimates generated from off-target reads by CopywriteR (Supplementary Fig. 2). The distribution of copy number loss detected by the two methods varied (Fig. 1A) and concurrent *BRCA1/BRCA2* copy number loss occurred more frequently by qPCR (5.6% v. 1.1%). Using CopywriteR as a benchmark, the sensitivity of qPCR as a method for detecting copy number events was poor (*BRCA1* 11.5%, *BRCA2* 36.8%, Fig. 1B). When grouping patients as CopywriteR *BRCA1* or *BRCA2* copy number loss versus copy number neutral, there was a significant difference in their *BRCA1* or *BRCA2* copy number value measured by qPCR (*BRCA1* *p* = 0.006, *BRCA2* *p* = 0.001, Fig. 1C). For those with *BRCA1* copy number loss by CopywriteR, the mean *BRCA1* qPCR copy number was 2.04 ± 0.69, versus 2.44 ± 0.90 (no loss). For *BRCA2*, the mean qPCR copy number was 1.32 ± 0.53 (copy number loss by CopywriteR) versus 1.87 ± 0.74 (no loss). However, the magnitude of correlation of copy number values measured by CopywriteR and qPCR was weak for both *BRCA1* (rho = 0.27, 95% CI 0.17–0.36) and *BRCA2* (rho = 0.29, 95% CI 0.20–0.39).

### *BRCA1*/*2* mRNA expression in *BRCA1/2* copy number loss cases (cohort 1)

Copy number data was integrated with existing *BRCA1*/*2* mutation (single nucleotide variant) calls and transcriptomic data. None of the *BRCA1*-mutant tumours (*n* = 46) demonstrated *BRCA1* copy number loss as determined by qPCR; within the *BRCA2*-mutant population, the frequency of *BRCA2* loss was 26% (6/23 cases). Within cases that did not demonstrate *BRCA1*/*2* mutation (*BRCA1*/*2* SNV wildtype), tumours with *BRCA1* copy number loss by qPCR did not demonstrate significantly lower *BRCA1* mRNA expression (*p* = 0.70). For *BRCA2*, *BRCA2* copy number lost tumours demonstrated lower *BRCA2* mRNA expression, but the difference did not pass the threshold for statistical significance (*p* = 0.06).

### Experimental factors influencing copy number loss detection by qPCR (cohort 1)

Experimental factors were examined for their potential to confound copy number assessment, particularly, the frequent occurrence of both *BRCA1* and *BRCA2* copy number loss by qPCR. Review of tumour cellularity demonstrated that the majority of samples had high tumour cellularity (47.3% with > 80%, 39.7% with 61–80% cellularity), and only 29 samples (8.2%) had a tumour cellularity of 40% or less (Supplementary Fig. 3). There was no significant difference in *BRCA1* or *BRCA2* qPCR copy number when comparing samples grouped by cellularity. Adjusting the copy number for cellularity did not significantly improve the sensitivity of qPCR as a method of copy number detection (*BRCA1;* sensitivity improvement 11.5% to 19.2% [95% CI 9.6–32.5], *BRCA2* 36.8% to 42.1% [95% CI 20.3–66.5]).

**Fig. 2:**
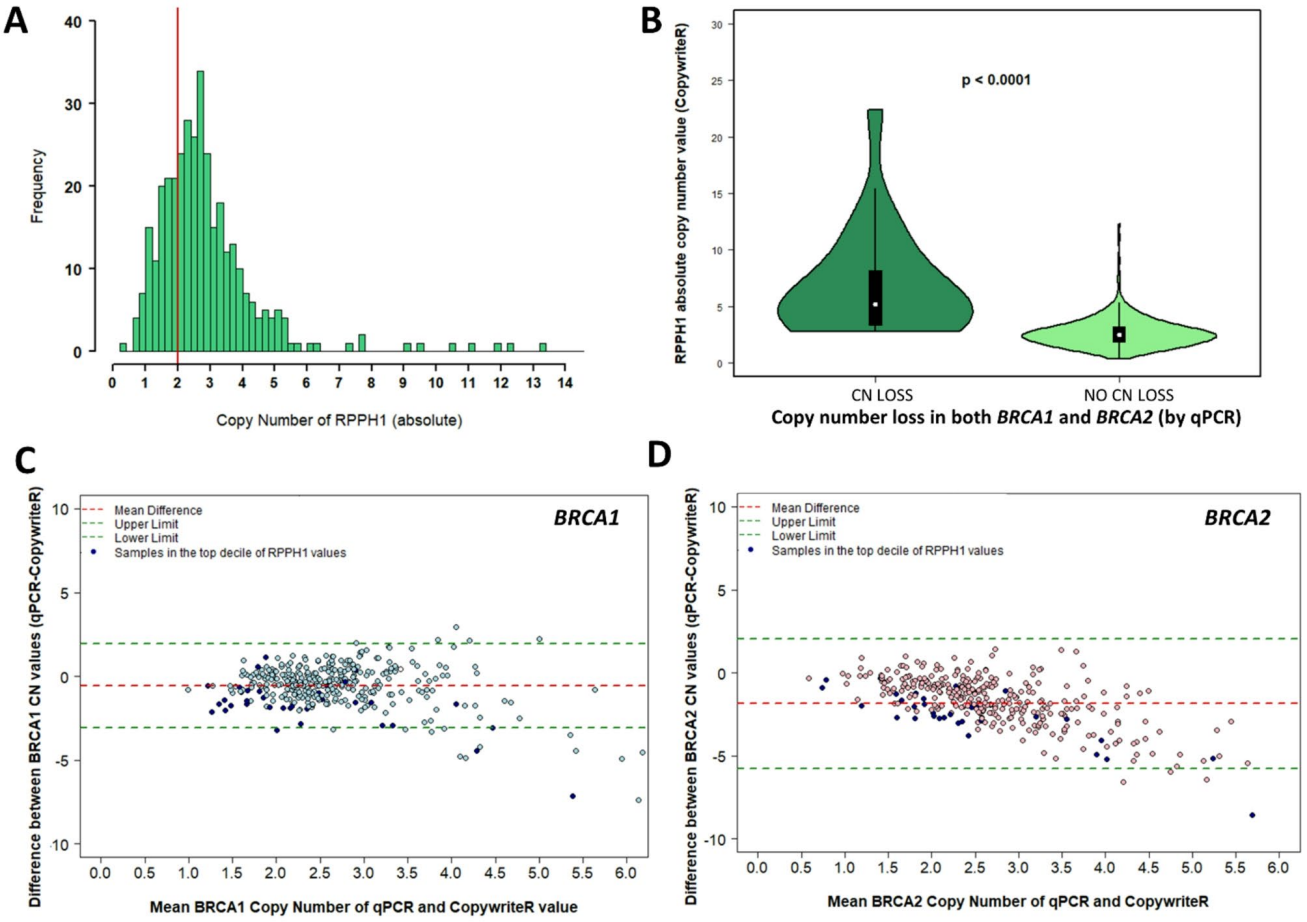
**(A)** Variation in copy number of reference probe *RPPH1* (RNaseP) by CopywriteR. Copy number normal (2 copies) is denoted by red line. **(B)** *RPPH1* copy number value (RNaseP) for samples with concurrent copy number loss by qPCR in *BRCA1* and *BRCA2*, versus samples without copy number loss. **(C**,** D)** Bland Altman plot of copy number values for *BRCA1* and *BRCA2* when comparing the two methods of copy number assessment (qPCR versus CopywriteR). Difference is copy number value from qPCR minus CopywriteR.

Next, copy number variation at the reference locus (RNaseP, *RPPH1*) was explored. *RPPH1* copy number values, extracted via CopywriteR, demonstrated a median copy number was 2.59 ± 1.96, with a range between copy number of 0.39 and 22.41 (Fig. 2A). A quarter of samples had an absolute *RPPH1* copy number of 3.5 or greater. *RPPH1* copy number was significantly higher in samples with concurrent *BRCA1* and *BRCA2* copy number loss by qPCR (median *RPPH1* copy number 5.20 ± 4.81 versus 2.48 ± 1.34, *p* < 0.0001, Fig. 2B). There were a high proportion of samples with a low mean copy number of *BRCA1/2* (mean of qPCR and CopywriteR value), that were noted to have a *RPPH1* copy number value within the top decile (Fig. 2C and D).

### qPCR performance in an independent cohort with whole genome sequencing (cohort 2)

**Fig. 3:**
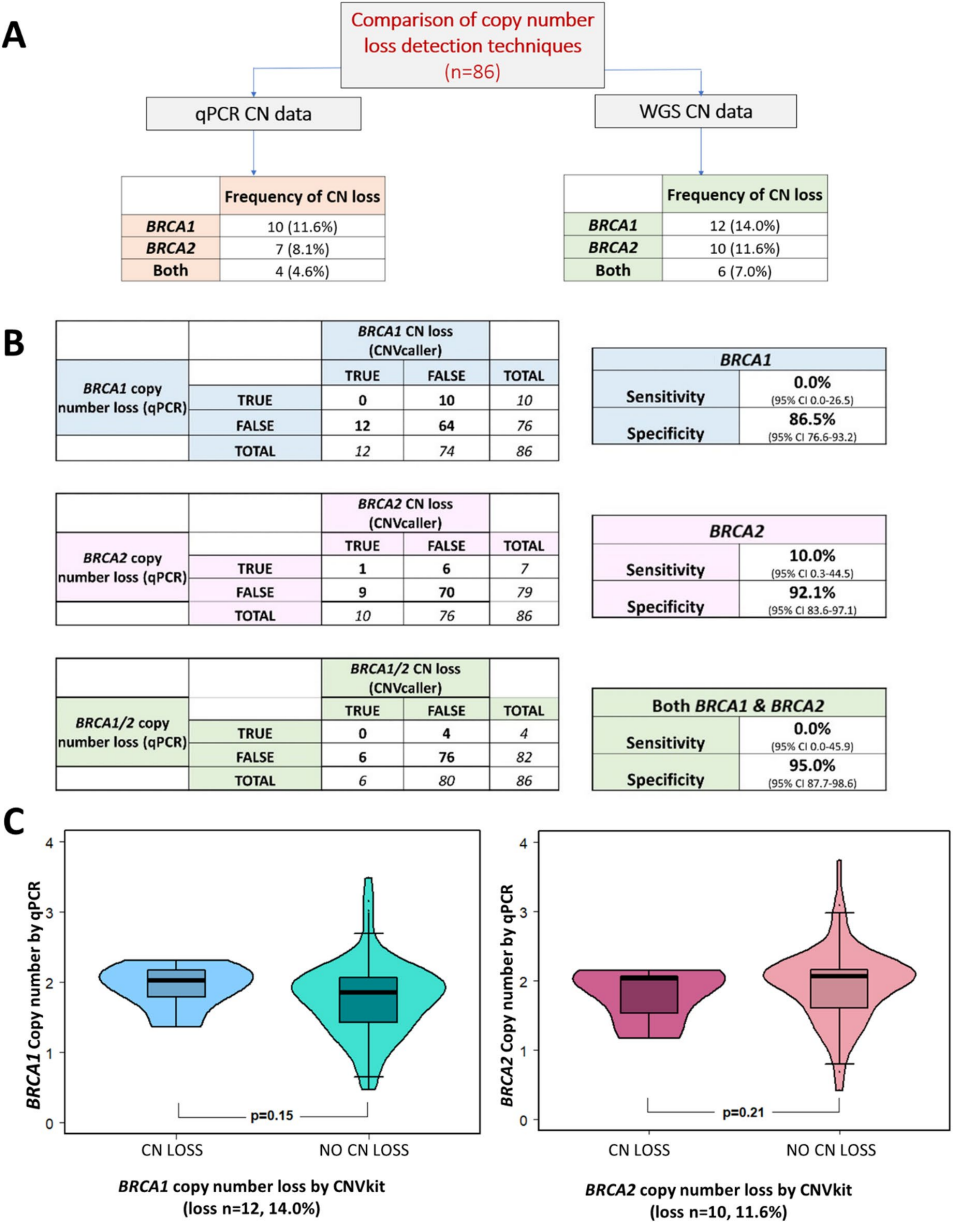
**(A)** Frequency of copy number loss events in cohort 2 (qPCR method versus CNVkit by WGS). **(B)** Sensitivity & Specificity of qPCR in identifying copy number loss events for *BRCA1*,* BRCA2* or both genes, compared with benchmark WGS/CNVkit. **(C)** Copy number value by qPCR, when samples were grouped as *BRCA1* or *BRCA2* copy number loss by WGS/CNVkit.

A second cohort of 86 HGSOC patients with available matched tumour-normal WGS was investigated using the same qPCR approach. Similar to cohort 1, the correlation for probes from each gene end was excellent (*BRCA1* rho = 0.94 [95% CI 0.91–0.96], *BRCA2* rho = 0.95 [95% CI 0.92–0.97]). The distribution of *BRCA1/2* copy number by qPCR was similar to that reported in the literature,^11^ with copy number loss of *BRCA1* occurring in 10 cases (11.6%) and in 7 cases (8.1%) for *BRCA2* (Fig. 3A). Compared to cohort 1, loss in both *BRCA1* and *BRCA2* occurred at a similar frequency (4.6% vs. 5.6%), but fewer *BRCA1* loss cases had concurrent *BRCA2* loss (cohort 1; 20/22 cases [91%], versus cohort 2; 4/10 cases [40%] of *BRCA1* loss also demonstrating *BRCA2* loss).

qPCR was compared to the *BRCA1/2* copy number data from CNVkit, derived from WGS data (Supplementary Fig. 4), with similar frequencies of *BRCA1/2* copy number loss in each cohort (Fig. 3A). Utilising CNVkit as a benchmark, qPCR demonstrated poor sensitivity as a method for identifying copy number loss events of *BRCA1/2* (sensitivity 0–10%, Fig. 3B). The correlation of copy number measured by qPCR and CNVkit was also poor (*BRCA1* rho = 0.21 [95% CI 0.01–0.4], *BRCA2* rho = 0.12 [95% CI 0.01–0.32]), with no significant difference in qPCR copy number when grouping samples based on their CNVkit copy number loss status (*BRCA1* *p* = 0.15, *BRCA2* *p* = 0.21, Fig. 3C).

**Fig. 4:**
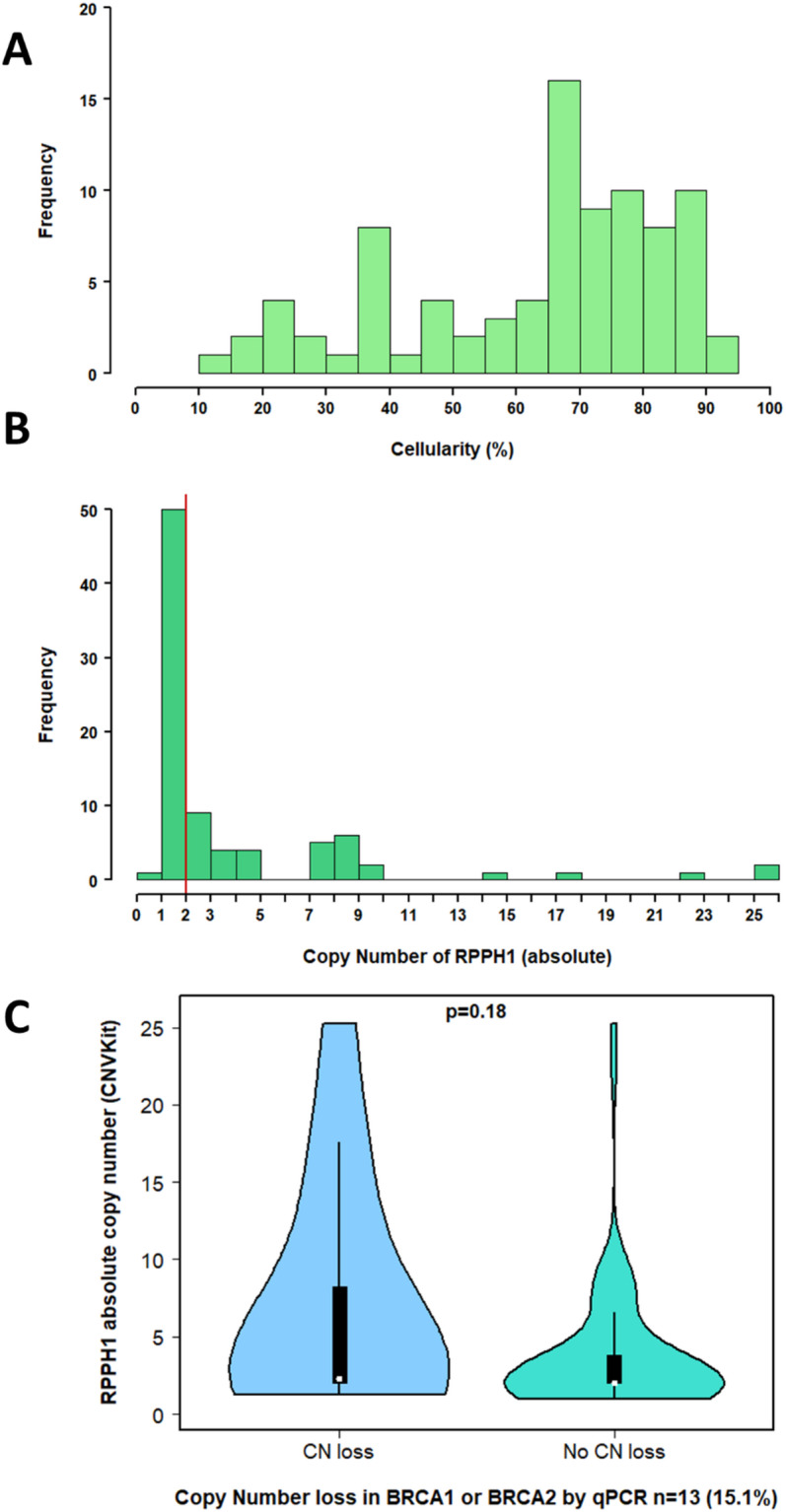
**(A)** Cellularity of samples in cohort 2 (*n* = 86). **(B)** Copy number variation of *RPPH1* (RNaseP) via CNVkit. Copy number normal (2 copies) is marked with the vertical red line. **(C)** Variation in *RPPH1* (RNaseP) copy number value (from CNVkit), in the group with and without copy number loss of either *BRCA1* or *BRCA2* (*n* = 13/86, 15.1%) by qPCR.

Experimental factors were also examined in this cohort. The median sample cellularity in cohort 2 was 70%; however, 24.4% of samples had a cellularity of less than 50% (Fig. 4A). There was no significant difference in *BRCA1* or *BRCA2* qPCR copy number when samples were grouped by cellularity (*BRCA1* *p* = 0.23, *BRCA2* *p* = 0.42). When copy number values were adjusted for cellularity, the improvement in sensitivity for detecting *BRCA1/2* copy number loss events was marginal (*BRCA1;* sensitivity improvement 0% to 8.33% [95% CI 0.21–38.5], *BRCA2* 10% to 20% [95% CI 2.52–55.6]). *RPPH1* copy number varied substantially between samples, with a median copy number of 2.0 ± 4.90 and a range of 0.98 to 25.23 (Fig. 4B). The subgroup identified as having qPCR copy number loss in either *BRCA1* or *BRCA2* (*n* = 17/86) demonstrated higher *RPPH1* copy number value (from CNVkit; mean copy number 6.8 ± 1.6 versus 3.6 ± 1.0), but this difference was not statistically significant (*p* = 0.18, Fig. 4C). Four samples had qPCR copy number loss of both *BRCA1* and *BRCA2*, with two of these demonstrating a markedly high *RPPH1* copy number (greater than 8 copies).

### qPCR performance in a HGSOC cell lines

**Fig. 5:**
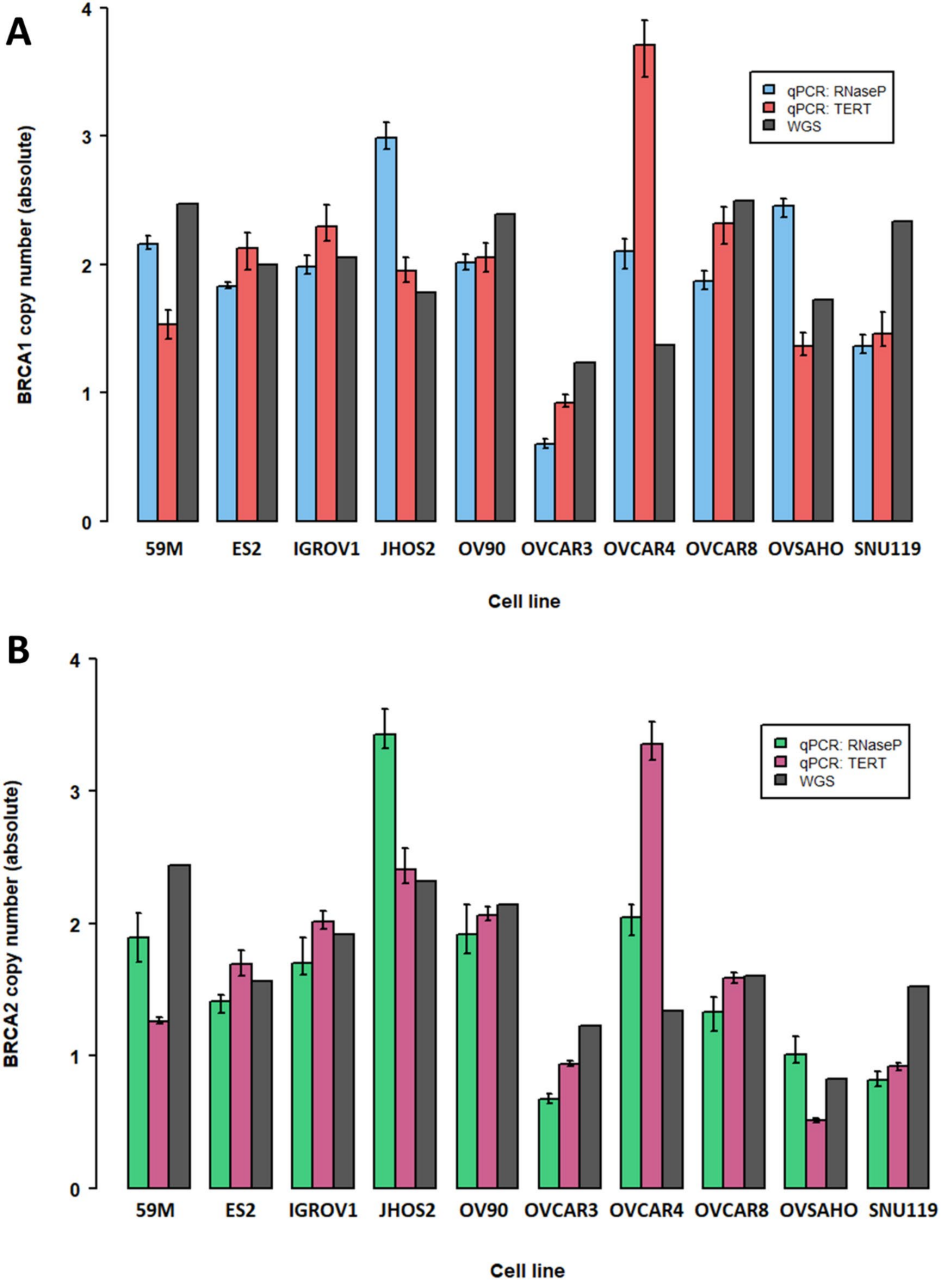
Copy number of *BRCA1* **(A)** or *BRCA2* **(B)** in a panel of HGSOC cell lines (*n* = 10) using qPCR and either reference probe (RNaseP/*RPPH1* or TERT), compared to *BRCA1* or *BRCA2* copy number extracted using copy number caller CNVkit from WGS data. qPCR values are the average from dual probing for *BRCA1* and *BRCA2* respectively with each reference assay, with error bars representing 95% confidence interval from three technical replicates.

*BRCA1/2* copy number was assessed by qPCR in a panel of HGSOC cell lines (*n* = 10), and compared to copy number derived from matched WGS (Fig. 5). Alongside the standard *RPPH1* reference assay, an additional reference locus (*TERT*) was implemented, due to the above described variation of *RPPH1* copy number identified in clinical samples.

**Fig. 6:**
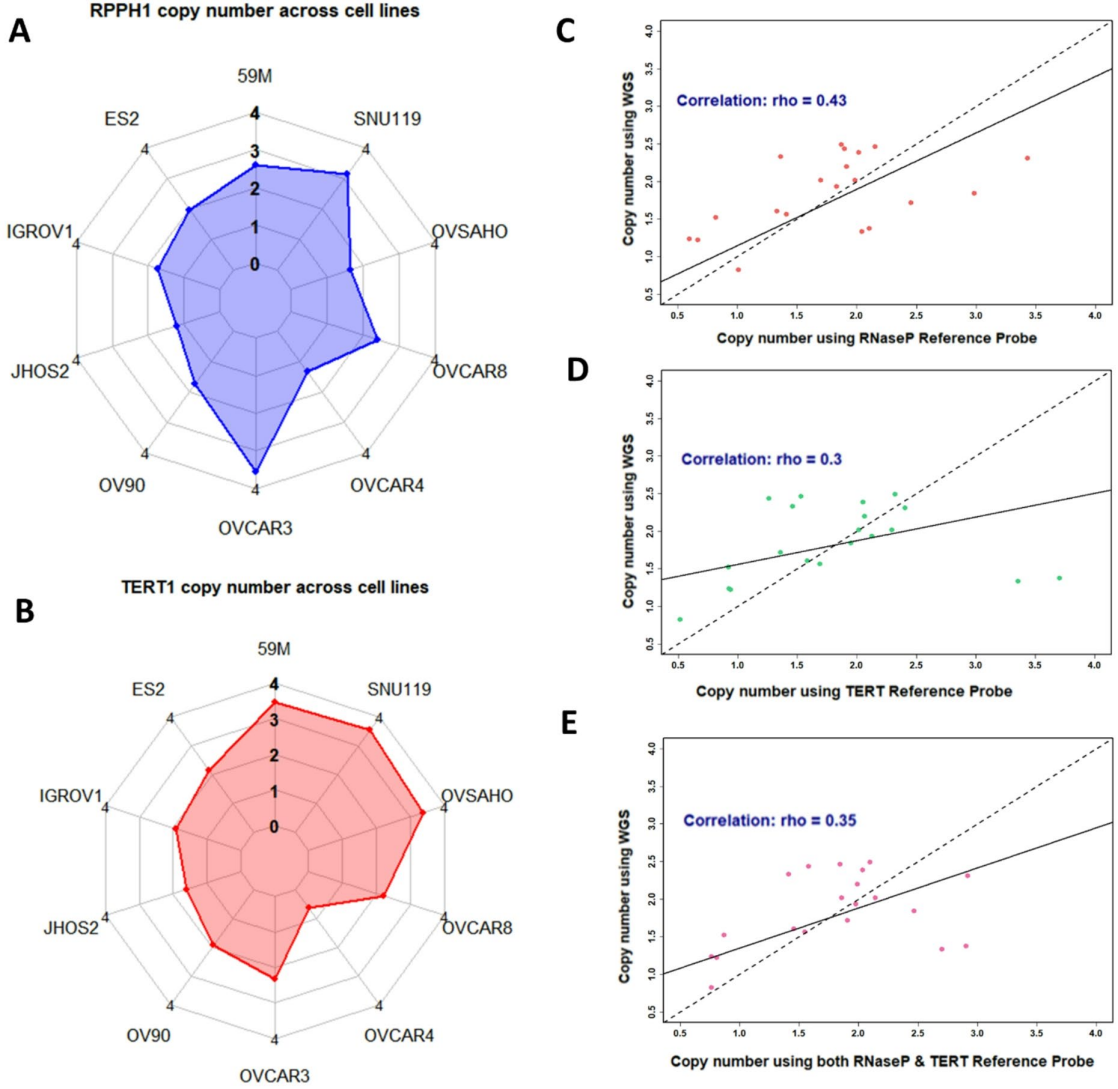
**(A**,** B)** Absolute copy number of reference assays (*RPPH1*, *TERT*) using copy number caller CNVkit from WGS in HGSOC cell lines (*n* = 10). Correlation (dotted line) between copy number of *BRCA1* and *BRCA2*, measured by qPCR versus WGS. **(C)** Correlation between qPCR using RNaseP as reference assay versus WGS. **(D)** Correlation between qPCR using TERT as reference assay versus WGS. **(E)** Correlation between qPCR using an average both reference assays for qPCR versus WGS. Dotted line represents a reference line (x = y).

When using RNaseP (*RPPH1*) as the reference probe and comparing qPCR to WGS copy number, the agreement was variable (Fig. 5). Half (5/10) of the cell lines (JHOS2, OVCAR3, OVCAR4, OVSAHO, SNU119) and 4/10 cell lines (JHOS2, OVCAR3, OVCAR4, SNU119) had a > 30% difference in *BRCA1* or *BRCA2* WGS copy number value, respectively. A consistent discrepancy pattern was observed for both *BRCA1* and *BRCA2*; for example, JHOS2, OVCAR4 and OVSAHO demonstrated an erroneously high copy number of both *BRCA1* and *BRCA2* by qPCR, whilst SNU119 and OVAR3 demonstrated an erroneously low copy number of both genes (Fig. 5). These discrepancies could be explained by copy number variation at the genomic co-ordinates of *RPPH1* in the WGS data (Fig. 6A). SNU119 and OVCAR3 demonstrated copy number gain (absolute copy number 3.1–3.5) at the site of *RPPH1*, whilst JHOS2, OVCAR4 and OVSAHO had *RPPH1* copy number loss (absolute copy number 1.2–1.6).

When using *TERT* as the qPCR reference probe, 3/10 cell lines (59 M, OVCAR4, SNU119) and 4/10 cell lines (59 M, OVCAR4, OVSAHO, SNU119) had a > 30% difference in *BRCA1* or *BRCA2* WGS copy number value, respectively (Fig. 5). Similarly to above, a consistent pattern for discrepancies was observed for both *BRCA1* and *BRCA2*. The copy number for both *BRCA1* and *BRCA2* by qPCR for OVCAR4 was significantly higher than expected based on WGS data (*BRCA1* copy number; qPCR 3.7 versus WGS 1.4, *BRCA2* qPCR 3.4 versus WGS 1.3), whilst the copy number for both *BRCA1* and *BRCA2* by qPCR for 59 M and SNU119 was significantly lower than expected (59M: *BRCA1* copy number; qPCR 1.5 versus WGS 2.5, *BRCA2*; qPCR 1.3 versus 2.4; SNU119: *BRCA1* copy number; qPCR 1.5 versus WGS 2.5, *BRCA2*; qPCR 1.9 versus 2.4). This variation was also in keeping with copy number alterations at the genomic co-ordinates of *TERT* (Fig. 6B); with the WGS *TERT* copy number being only 0.6 in OVCAR4 and elevated in 59 M (3.5), SNU119 (3.6) and OVSAHO (3.4).

The correlation between copy number measured by qPCR using either reference probe (*RPPH1* or *TERT*), versus copy number measured by WGS was poor (qPCR using *RPPH1* rho = 0.43, qPCR using *TERT* rho = 0.30, Fig. 6C-E). Utilising both reference probes (as an average) did not improve the correlation between qPCR copy number versus WGS copy number (rho = 0.35).

## Discussion

The aim of this study was to evaluate whether qPCR, using readily available commercial assays and established reference probes, could serve as a simple and reliable method for identifying *BRCA1* or *BRCA2* copy number loss in HGSOC. Given the established phenotypic significance of *BRCA1/2* copy number loss^11,12,14^, this question has important clinical relevance, and qPCR offers the potential to overcome the limitations of alternative copy number analysis methods. No previous multi-cohort study has evaluated qPCR as a method for *BRCA1/2* copy number loss detection. Utilising sequencing-based approaches as a benchmark, qPCR had a low sensitivity in detecting *BRCA1/2* copy number loss in two independent HGSOC patient samples. Though co-deletion of *BRCA1* and *BRCA2* has been described (in around 3% of cases)^11^, the high rate of concurrent copy number loss among *BRCA1* and *BRCA2* raised potential concerns regarding the reference probe, which was supported by significant variation of *RPPH1* copy number from matched sequencing data.

For cohort 1, sensitivity analysis suggested a suboptimal performance for copy number loss detection by qPCR (sensitivity rate *BRCA1* 11.5%, *BRCA2* 36.8%). However, this analysis was reliant on absolute thresholds, which have not been validated. Grouping samples as copy number loss versus no loss did demonstrate a significant difference in qPCR copy number (*BRCA1* *p* = 0.006, *BRCA2* *p* = 0.001), suggesting some overlap between methods. Though this cohort was large and well annotated, it does have limitations in relation to this study. It utilises CopywriteR, which leverages off target reads to overcome exon distribution and non-uniform bait efficiencies.^19^ Though this allows effective copy number analysis; performance is contingent on the efficiency of the target enrichment process and read depth.^19^ Furthermore, these samples were extracted from FFPE material; formalin fixation markedly impacts nucleic acid integrity^25^ and DNA crosslinking from fixation may impact accessibility of qPCR assays.^26^ Cohort 2 offered higher quality sequencing data (WGS) obtained from fresh frozen tissue, and also demonstrated that qPCR had poor sensitivity in detecting *BRCA1/2* copy number loss events (sensitivity rate *BRCA1* 0.0%, *BRCA2* 10.0%). It utilised CNVkit, which calculates genome-wide copy ratios from both on- and off-target reads, primarily leveraging read depth with additional paired-end split-read information to improve accuracy.^21^ This cohort was however limited by lower tumour cellularity percentages and a smaller sample size.

The accuracy of utilising qPCR for detecting *BRCA1/2* copy number loss was assessed in a third cohort, using HGSOC cell lines. Cell line DNA eradicated concerns regarding tumour cellularity and intratumour heterogeneity, which are limitations with any clinical cohort. This work further demonstrated inconsistencies in the measurement of *BRCA1/2* copy number by qPCR, compared to WGS copy number values. Despite it being common practice to have one reference assay for qPCR for copy number analysis^24^, an additional commercially available reference assay (*TERT*) was also included in this cell line cohort^27^ due to the concerns raised regarding the single standard reference probe *RPPH1* in the clinical cohorts. *TERT* is considered the alternative assay, as there is evidence that *TERT* is frequently amplified in multiple cancer types.^28^ Neither reference assay appeared superior, and using both reference assays did not improve the correlation between copy number values measured by qPCR versus WGS. Furthermore, variation in the copy number of *RPPH1* and *TERT*, derived from cell line WGS data, provided an explanation for the variations in *BRCA1/2* copy number between reference assays. This was highly supportive of the existing concerns that reference assay copy number variation was contributing to the poor qPCR sensitivity in clinical samples.

This work makes a valuable contribution to the field, as few studies have directly evaluated qPCR for copy number analysis, and existing studies have primarily focused on amplification events. In HGSOC, qPCR has been used to demonstrate biologically and clinically distinct subgroups associated with copy number gain of *EMSY* and *CCNE1*.^9^ However, a greater fold difference to normal was utilised to define gain (in comparison to this fold difference for loss), and reference probe amplification in this context would result in under-reporting gain events (thereby affecting specificity rather than sensitivity). In other tumour types, one study validated qPCR on FFPE samples across multiple cancer genes and demonstrated good concordance with sequencing; however, this study had a smaller sample size, focused on amplifications and included genomically stable tumour types.^29^ Few studies have evaluated qPCR for copy number loss; one study of *CDK2NA* loss in gastric cancer samples reported good correlation with sequencing copy number data, but poor sensitivity and specificity.^30^.

Though our data focused on *BRCA1/2* loss, there is growing evidence of the biological and clinical importance of loss in other tumour suppressor genes,^3,10^ suggesting broader relevance of this work. For instance, loss of *CDK2NA* has demonstrated importance in terms of prognostication and treatment response in a range of tumour types such as bladder, gastric, lung and head and neck cancers.^30–33^ Furthermore, *PTEN* loss is a recognised biomarker for therapeutic stratification in breast cancer (e.g. capivasertib).^34^ These observations underscore the need for accurate and accessible methods to detect copy number loss, which could have immediate clinical implications across a range of tumour types. While our findings suggest that qPCR is not a reliable approach for detecting *BRCA1/2* copy number loss in HGSOC, its utility in other tumour types (particularly those less prone to genomic variation at the reference assay sites), remains to be determined.

## Conclusion

Analysis of two independent clinical datasets and a panel of cell lines indicated that qPCR using standard assays could not reliably detect *BRCA1* or *BRCA2* copy number loss in HGSOC. Copy number variation of the copy number reference locus is a likely confounding factor that can result in samples being erroneously identified as having copy number loss. Overall, these findings indicate the need for caution when using qPCR as a method for identifying copy number loss events. This is of particular importance in cancers such as HGSOC which is typified by genomic instability and widespread copy number alterations. Currently, sequencing based detection of SVs appears to represent the most accurate method for identifying patients with tumours harbouring *BRCA1/2* copy number loss, with WGS being the optimal method to characterise *BRCA1/2* SVs.

## Supplementary Information

Below is the link to the electronic supplementary material.

Supplementary Material 1

## Data Availability

WGS from the Scottish cohort (SHGSOC) is available via EGA at accession code EGAS00001004410. All other data are available upon reasonable request to the corresponding author, subject to requests falling within our local ethics framework.

## References

[1] Hamdan, A. & Ewing, A. Unravelling the tumour genome: the evolutionary and clinical impacts of structural variants in tumourigenesis. *J Pathol Jul*. **257** (4), 479–493. 10.1002/path.5901 (2022). 10.1002/path.5901 PMC9321913 35355264

[2] Zhao, M. & Zhao, Z. Concordance of copy number loss and down-regulation of tumor suppressor genes: a pan-cancer study. *BMC Genomics Aug*. **22** (Suppl 7), 532. 10.1186/s12864-016-2904-y (2016). 10.1186/s12864-016-2904-y PMC5001246 27556634

[3] Besedina, E. & Supek, F. Copy number losses of oncogenes and gains of tumor suppressor genes generate common driver mutations. *Nature Communications*. **15**(1), 6139 10.1038/s41467-024-50552-1 (2024). 10.1038/s41467-024-50552-1 PMC11271286 39033140

[4] Scott, A. J., Chiang, C. & Hall, I. M. Structural variants are a major source of gene expression differences in humans and often affect multiple nearby genes. *Genome Res Dec.* **31** (12), 2249–2257. 10.1101/gr.275488.121 (2021). 10.1101/gr.275488.121 PMC8647827 34544830

[5] van Belzen, I. A. E. M., Schönhuth, A., Kemmeren, P. & Hehir-Kwa, J. Y. Structural variant detection in cancer genomes: computational challenges and perspectives for precision oncology. *npj Precision Oncology*. **5**(1), 15. 10.1038/s41698-021-00155-6 (2021). 10.1038/s41698-021-00155-6 PMC7925608 33654267

[6] Theik, N. W. Y. et al. NTRK therapy among different types of Cancers, review and future perspectives. *Int J. Mol. Sci Feb*. **17** (4). 10.3390/ijms25042366 (2024). 10.3390/ijms25042366 PMC10889397 38397049

[7] Rakha, E. A. et al. UK recommendations for HER2 assessment in breast cancer: an update. *J Clin. Pathol Apr*. **76** (4), 217–227. 10.1136/jcp-2022-208632 (2023). 10.1136/jcp-2022-208632 36564170

[8] Etemadmoghadam, D. et al. Integrated genome-wide DNA copy number and expression analysis identifies distinct mechanisms of primary chemoresistance in ovarian carcinomas. *Clin Cancer Res Feb*. **15** (4), 1417–1427. 10.1158/1078-0432.Ccr-08-1564 (2009). 10.1158/1078-0432.CCR-08-1564 PMC2670486 19193619

[9] Hollis, R. L. et al. Multiomic characterization of High-Grade serous ovarian carcinoma enables High-Resolution patient stratification. *Clin Cancer Res Aug*. **15** (16), 3546–3556. 10.1158/1078-0432.Ccr-22-0368 (2022). 10.1158/1078-0432.CCR-22-0368 PMC9662902 35696721

[10] Wee, Y., Wang, T., Liu, Y., Li, X. & Zhao, M. A pan-cancer study of copy number gain and up-regulation in human oncogenes. *Life Sciences*. **211, **206–214. 10.1016/j.lfs.2018.09.032 (2018). 10.1016/j.lfs.2018.09.032 30243646

[11] Ewing, A. et al. Structural variants at the BRCA1/2 loci are a common source of homologous repair deficiency in high grade serous ovarian carcinoma. *Clin. Cancer Res.* **clincanres.CCR-20-4068-A.2020**10.1158/1078-0432.CCR-20-4068 (2021). 10.1158/1078-0432.CCR-20-4068 PMC7610896 33741650

[12] Swisher, E. M. et al. Characterization of patients with long-term responses to Rucaparib treatment in recurrent ovarian cancer. *Gynecol Oncol Dec.* **163** (3), 490–497. 10.1016/j.ygyno.2021.08.030 (2021). 10.1016/j.ygyno.2021.08.030 34602290

[13] Nguyen, L., Martens, W. M., Van Hoeck, J. & Cuppen, A. E. Pan-cancer landscape of homologous recombination deficiency. *Nature Communications*. **11**(1), 5584. 10.1038/s41467-020-19406-4 (2020). 10.1038/s41467-020-19406-4 PMC7643118 33149131

[14] Oswald, A. et al. 350 (PB338): structural variants of BRCA1/2 represent a novel biomarker of homologous recombination deficiency in multiple tumour types. *Eur. J. Cancer*. **211**. 10.1016/j.ejca.2024.114863 (2024).

[15] Nukaya, T. et al. Estimating copy number to determine BRCA2 deletion status and to expect prognosis in localized prostate cancer. *Cancer Med Jan*. **16**10.1002/cam4.5617 (2023). 10.1002/cam4.5617 PMC10134377 36645189

[16] Kluth, M. et al. 13q deletion is linked to an adverse phenotype and poor prognosis in prostate cancer. *Genes Chromosomes Cancer Oct.* **57** (10), 504–512. 10.1002/gcc.22645 (2018). 10.1002/gcc.22645 29923647

[17] Chakraborty, G. et al. Significance of BRCA2 and RB1 Co-loss in aggressive prostate cancer progression. *Clin Cancer Res Apr*. **15** (8), 2047–2064. 10.1158/1078-0432.Ccr-19-1570 (2020). 10.1158/1078-0432.CCR-19-1570 PMC7416644 31796516

[18] Pös, O. et al. Copy number variation: methods and clinical applications. *Appl. Sci.* **11** (2), 819 (2021).

[19] Kuilman, T. et al. CopywriteR: DNA copy number detection from off-target sequence data. *Genome Biol Feb*. **27** (1), 49. 10.1186/s13059-015-0617-1 (2015). 10.1186/s13059-015-0617-1 PMC4396974 25887352

[20] Ewing, A. et al. Divergent trajectories to structural diversity impact patient survival in high grade serous ovarian cancer. *Nat Commun Jul*. **1** (1), 5586. 10.1038/s41467-025-60655-y (2025). 10.1038/s41467-025-60655-y PMC12215056 40593568

[21] Talevich, E., Shain, A. H., Botton, T., Bastian, B. C. & CNVkit Genome-Wide copy number detection and visualization from targeted DNA sequencing. *PLoS Comput. Biol Apr*. **12** (4), e1004873. 10.1371/journal.pcbi.1004873 (2016). 10.1371/journal.pcbi.1004873 PMC4839673 27100738

[22] Capes-Davis, A. et al. Match criteria for human cell line authentication: where do we draw the line? *Int J. Cancer Jun*. **1** (11), 2510–2519. 10.1002/ijc.27931 (2013). 10.1002/ijc.27931 23136038

[23] Ghandi, M. et al. Next-generation characterization of the cancer cell line encyclopedia. *Nature May*. **569** (7757), 503–508. 10.1038/s41586-019-1186-3 (2019). 10.1038/s41586-019-1186-3 PMC6697103 31068700

[24] Mayo, P. et al. CNV analysis using TaqMan copy number assays. *Curr Protoc Hum Genet*. Oct 2010;Chap. 2:Unit2.13. 10.1002/0471142905.hg0213s67 10.1002/0471142905.hg0213s67 20891030

[25] Gao, X. H. et al. Comparison of fresh frozen tissue with Formalin-Fixed Paraffin-Embedded tissue for mutation analysis using a Multi-Gene panel in patients with colorectal cancer. *Front. Oncol.* **10**, 310. 10.3389/fonc.2020.00310 (2020). 32232001 10.3389/fonc.2020.00310 PMC7083147

[26] Cantsilieris, S., Baird, P. N. & White, S. J. Molecular methods for genotyping complex copy number polymorphisms. *Genomics Feb*. **101** (2), 86–93. 10.1016/j.ygeno.2012.10.004 (2013). 10.1016/j.ygeno.2012.10.004 23123317

[27] D’Haene, B., Vandesompele, J. & Hellemans, J. Accurate and objective copy number profiling using real-time quantitative PCR. *Methods Apr*. **50** (4), 262–270. 10.1016/j.ymeth.2009.12.007 (2010). 10.1016/j.ymeth.2009.12.007 20060046

[28] Colebatch, A. J., Dobrovic, A. & Cooper, W. A. TERT gene: its function and dysregulation in cancer. *J. Clin. Pathol.* **72** (4), 281. 10.1136/jclinpath-2018-205653 (2019). 30696697 10.1136/jclinpath-2018-205653

[29] Mehrotra, M. et al. Validation of quantitative PCR-based assays for detection of gene copy number aberrations in formalin-fixed, paraffin embedded solid tumor samples. *Cancer Genet.* **212–213**, 24–31. 10.1016/j.cancergen.2017.03.002 (2017). 2017/04/01/. 28449808 10.1016/j.cancergen.2017.03.002

[30] Tian, Y. et al. Detection of somatic copy number deletion of the CDKN2A gene by quantitative multiplex PCR for clinical practice. Original Research. *Frontiers in oncology*. *2022-December-02* 12. 10.3389/fonc.2022.1038380 (2022). 10.3389/fonc.2022.1038380 PMC9755846 36531022

[31] Papadimitriou, M-A. et al. CDKN2A copy number alteration in bladder cancer: integrative analysis in patient-derived xenografts and cancer patients. *Mol. Therapy: Oncol.* **32** (2), 200818. 10.1016/j.omton.2024.200818 (2024). 2024/06/20/. 10.1016/j.omton.2024.200818 PMC11223115 38966038

[32] Chen, W. S. et al. CDKN2A copy number loss is an independent prognostic factor in HPV-Negative head and neck squamous cell carcinoma. *Front. Oncol.* **8**, 95. 10.3389/fonc.2018.00095 (2018). 29670856 10.3389/fonc.2018.00095 PMC5893829

[33] Gutiontov, S. I. et al. CDKN2A loss-of-function predicts immunotherapy resistance in non-small cell lung cancer. *Scientific Reports*. **11**(1), 20059. 10.1038/s41598-021-99524-1 (2021). 10.1038/s41598-021-99524-1 PMC8501138 34625620

[34] Smyth, L. M. et al. Selective AKT kinase inhibitor Capivasertib in combination with fulvestrant in PTEN-mutant ER-positive metastatic breast cancer. *NPJ Breast Cancer Apr*. **16** (1), 44. 10.1038/s41523-021-00251-7 (2021). 10.1038/s41523-021-00251-7 PMC8052445 33863913

