# Supplementary material for: Evaluation of quantitative polymerase chain reaction for detecting BRCA1 or BRCA2 copy number loss in high-grade serous ovarian cancer
Source: Sci Rep. 2026 Jan 10;16:4374. doi: 10.1038/s41598-025-34516-z (PMC12864745; doi:10.1038/s41598-025-34516-z)
Supplement: Supplementary file 1 — Supplementary Material 1 [file 41598_2025_34516_MOESM1_ESM.docx]

**Supplementary methods:**

Gene Panel for cohort 1:

Sequencing was performed using a custom Integrated DNA Technologies (IDT) gene capture panel with unique molecular indices (UMIs) as described by Hollis et *al.* The gene target panel was designed to capture all exonic regions of: *ABCB1, AC004223.3, ARID1A, ATM, ATR, ATRX, BAP1, BARD1, BCL2L1, BLM, BRAF, BRCA1, BRCA2, BRIP1, C11orf65, CCNE1, CDK12, CHD4, CHEK1, CHEK2, CTNNB1, EGFR, EMSY, ERBB2, ERCC4, EZH2, FANCA, FANCB, FANCC, FANCD2, FANCE, FANCF, FANCG, FANCI, FANCL, FANCM, GNAS, KIT, KRAS, MAD2L2, MDM2, MLH1, MRE11, MSH2, MSH6, MUS81, MUTYH, NBN, NDUFB2, NF1, NF2, NRAS, PALB2, PARP1, PARP2, PAXIP1, PDGFRA, PER3, PIK3CA, PMS2, PPP2R1A, PPP2R2A, PRKDC, PTEN, RAD50, RAD51, RAD51B, RAD51C, RAD54L, RB1, RNASEH2A, RNASEH2B, RNASEH2C, RPA1, RUNDC3B, SHFM1, SLC25A40, SLFN11, SLX4, TOE1, TP53, TP53BP1, UBE2T, VRK2.*

Bioinformatic processing for cohort 1 (CopyrighteR):

For cohort 1, copy number analysis was performed using CopywriteR, whereby off-target reads were used to estimate the relative copy number of 50 kilobase segments across each chromosome from the aligned BAM files produced by the bcbio-nextgen workflow. For the present study, the available copy number estimates were processed in R (version 4.3.2), utilising the co-ordinates of *RPPH1* (Chr14: 20,343,075 – 20,343,407), *BRCA1* (Chr17: 43,044,295 –43,170,245) and *BRCA2* (Chr13: 32,315,086 – 32,400,268) from Ensembl (version 111).^20^ Given that the CopywriteR estimates represent quantified copy number across 50 kilobase segments, multiple segments encompassing the *BRCA1* or *BRCA2* genes were included and the mean of these values was used. Segments were included if there was at least 5kb of the gene located within the 50kb segment. Log2 copy number ratios from CopywriteR were converted to absolute copy number values by the following equation; (2^log2 ratio)/2. Threshold for copy number loss from CopywriteR values was heterozygous loss with a 20% tolerance (absolute copy number 1.2).

Bioinformatic processing for cohort 2 and cell lines (WGS, CNVkit):

For cohort 2, samples were whole genome sequenced on the HiSeq X Ten Illumina NovaSeq 6000. Reads were aligned to GRCh38, and copy number assessed using CNVkit^22^(version 0.9.3), using matched normal blood samples as reference. Log2 copy number ratios from CNVkit were converted to absolute copy number values by the following equation; (2^log2 ratio)/2.

The HGSOC cell lines and reference cell lines had been previously sequenced by the Broad Institute, on Illumina HiSeq 2000^24^, with publicly available WGS files. Raw FASTQ WGS files were downloaded via SRA-explorer and HCC1143-BL was used as a matched normal reference.^25^ FASTQ files were processed utilising bcbio-nextgen high throughput approaches, by the same pipeline as described for cohort 2, using HCC11430BL as the reference dataset. Data were aligned to the GRCh38 human reference genome to generate BAM files using bwa-0.7.17^26^, duplicates marked and base quality scores recalibrated with the GenomeAnalysisToolkit (version 4)^27^ within the bcbio 1.0.6 pipeline. CNV analysis was carried out using CNVkit (version 0.9.3).^22^

TaqMan PCR:

Copy number of *BRCA1/2* was quantified using TaqMan genotyping qPCR copy number assays, utilising 10ng template DNA as determined by HS qubit assay. VIC dye-labelled RNaseP copy number reference assay (target; Chr.14:20343370, catalog number 4403328, thermofisher scientific) was used alongside FAM labelled probes targeting *BRCA1* or *BRCA2*. Additional VIC dye-labelled copy number reference assay TERT was included in qPCR for the cell line cohort (target; Chr 5:1253373, catalog number 4403315, thermofisher scientific). For *BRCA1* and *BRCA2*, two probes were used for each gene respectively, to identify copy number alterations at both ends of the gene (*BRCA1*; intron 3 Hs06421688_cn, intron 20 Hs06422382_cn, thermofisher scientific) and *BRCA2* (intron 2; Hs05307796_cn, intron 24; Hs06368818_cn, thermofisher scientific). Efficiency calculation was performed on all assays confirming >95% efficiency, using the gradient of the line or best fit for Ct value, against the log (base 10) of ng DNA input (efficiency = -1 + 10-1/slope of Ct versus log10 of input DNA).^41^ NA12878 human reference DNA was purchased from the Coriell Institute and included in each plate. Plates were ran using the StepOne Plus Real-Time PCR System (Applied Biosystems, Thermo Fisher Scientific, Waltham, MA, USA), which was 95°C for 10 minutes, followed by 40 cycles of 95°C for 15 seconds, 60°C for 60 seconds. This was analysed and exported using the StepOne Software Version 2.3 (Applied Biosystems, Thermo Fisher Scientific, Waltham, MA, USA). Copy number variants were called using CopyCaller version 2.1 (Life Technologies, Applied Biosystems, Thermo Fisher Scientific, Waltham, MA, USA) using NA12878 as a calibrator sample with normal copy number (CN=2). Copy number of *BRCA1* or *BRCA2* was the mean of the copy number value from each probe at either end of the respective gene. Copy number loss was defined as a mean copy number of 1.2 or less, which was heterozygous copy number loss with a 20% tolerance for experimental variation due to tumour heterogeneity and normal tissue contamination. DNA from cell lines with known *BRCA1* or *BRCA2* copy number loss (as above), and DNA from a normal tissue sample were also included in each experimental run.

**Supplementary Figures**


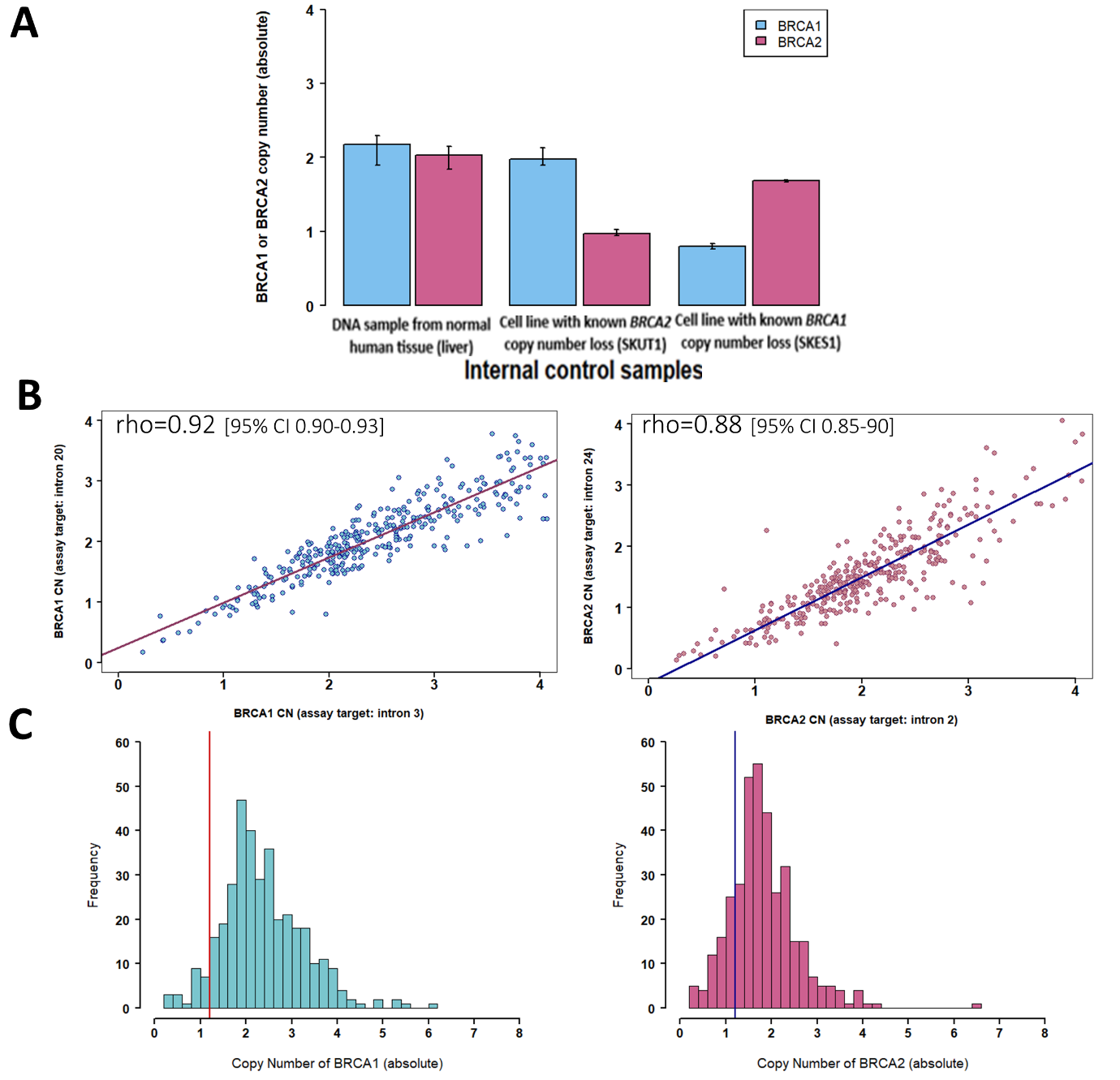


**Supplementary Figure 1. (A)** Copy number (BRCA1 and BRCA2) of internal control samples by qPCR, including normal human tissue sample and two known sarcoma cell lines with BRCA1 or BRCA2 copy number loss. Values are the average from dual probing for BRCA1 and BRCA2 respectively, with error bars representing 95% confidence interval from three technical replicates. These samples were included on every qPCR plate. **(B)** Correlation between two BRCA1 assays and two BRCA2 assays measuring copy number by qPCR at each end of the respective gene in Cohort 1. **(C)** Distribution of BRCA1/2 absolute copy number by qPCR in Cohort 1. The red (BRCA1) and blue (BRCA2) line indicates the threshold for considering copy number loss (absolute copy number of 1.2).


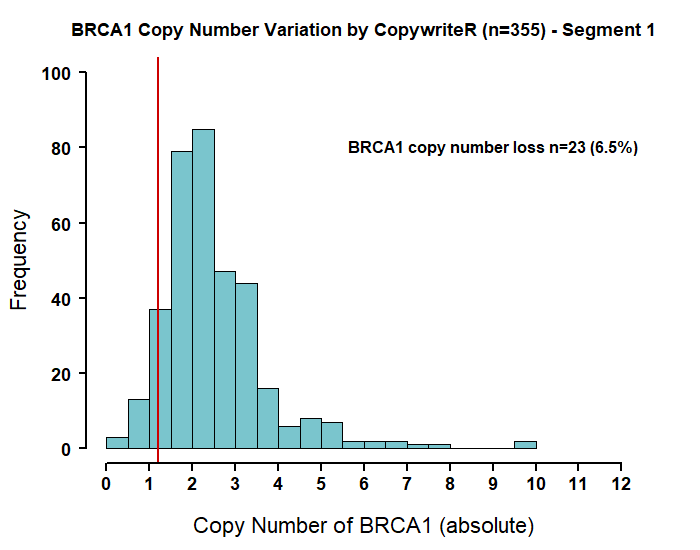

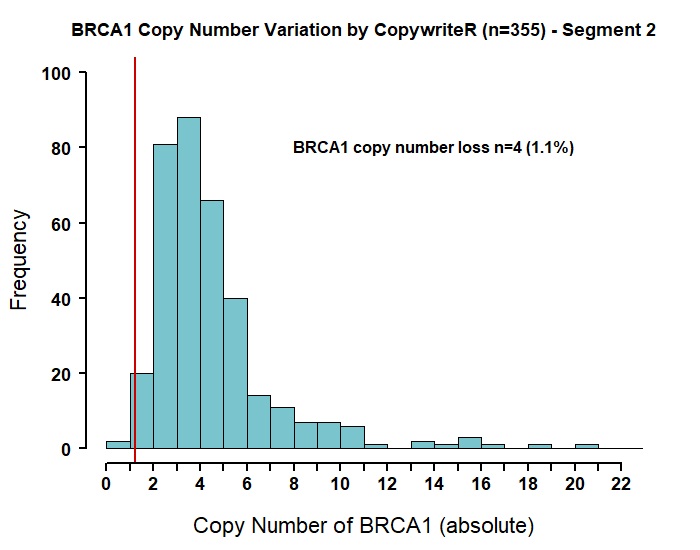

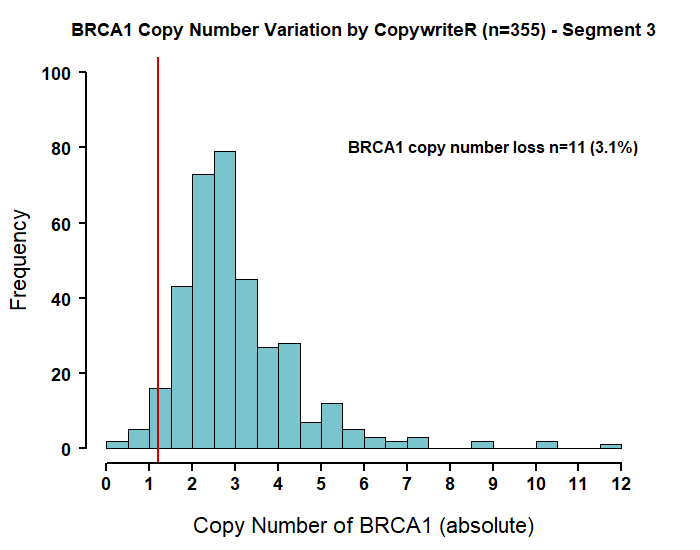

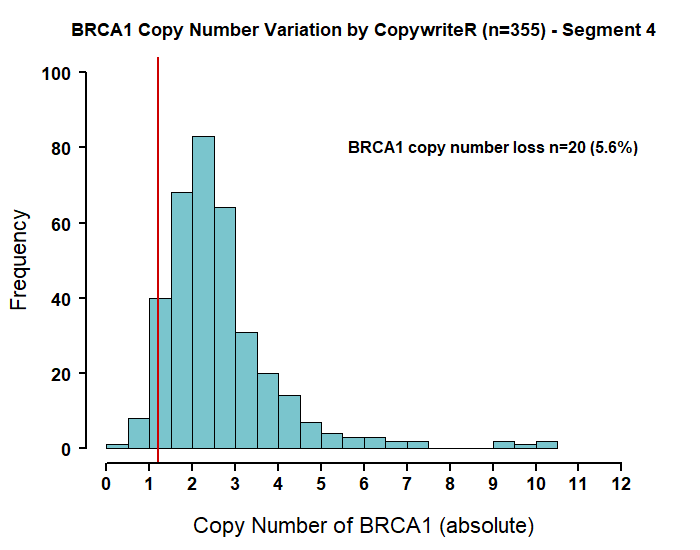

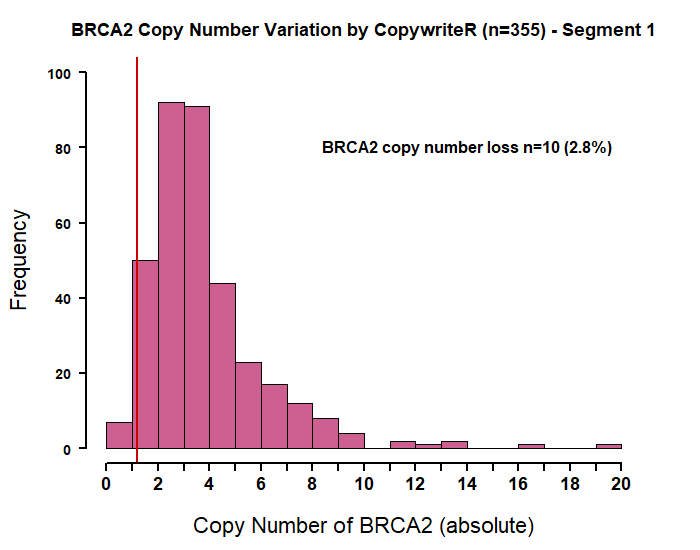

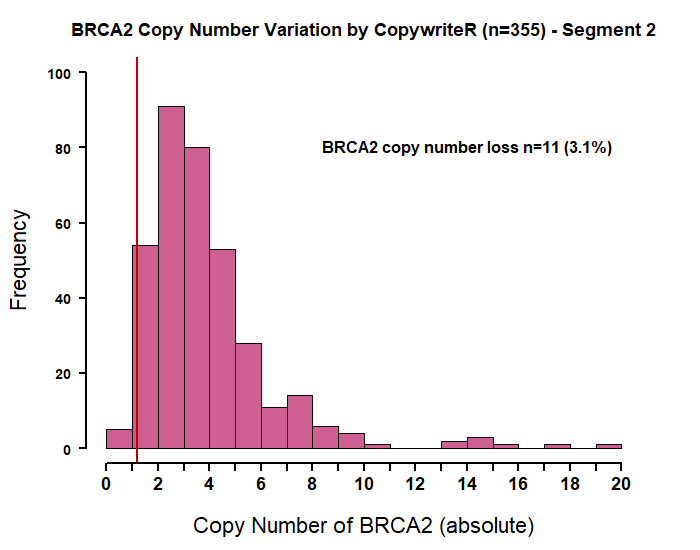


**Supplementary Figure 2**. Copy number variation at each segment of *BRCA1* and *BRCA2* by CopywriteR with frequency of copy number loss at each 50 kilobase segment in Cohort 1. The red (*BRCA1*) and blue (*BRCA2*) line indicates the threshold for considering copy number loss (absolute copy number of 1.2). For *BRCA1*, there were four 50kb pair segments included to encompass the entire gene, and for *BRCA2* there were two segments. Samples were considered to have copy number loss if any genomic 50kb segment had evidence of hemizygous copy number loss. ). The median copy number was 2.73 ± 1.18 for *BRCA1* and 3.23 ± 2.03 for *BRCA2*. For *BRCA1*, there were 52 (14.6%) of samples demonstrating copy number loss at any of the segments over the gene and 19 (5.4%) demonstrating copy number loss at any of the *BRCA2* gene segments. For *BRCA1*, no samples had loss of three or four segments, with only 6/52 (11.5%) having loss of two segments and the remaining 46/52 (88.5%) having loss of only a single segment. For *BRCA2*, 2/19 (10.5%) samples had loss of both segments and 17/19 (89.5%) had loss of one segment.

**A**

**
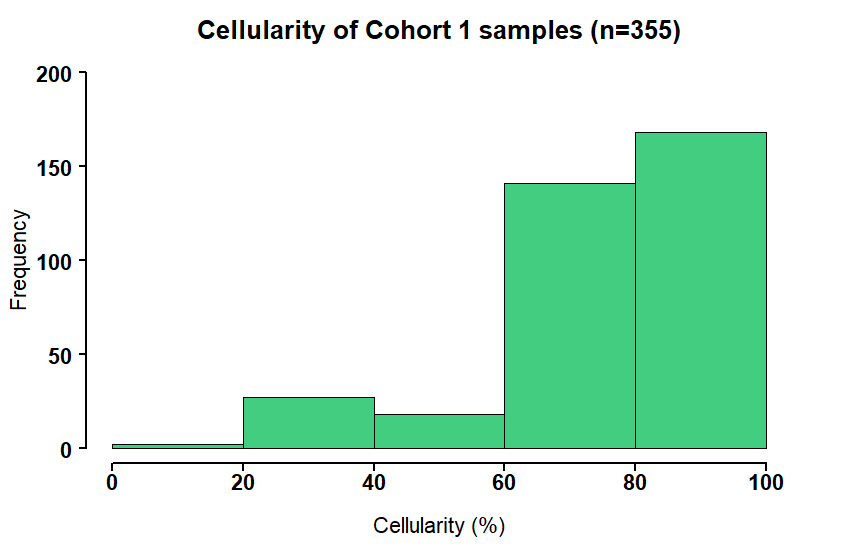
**

**B**


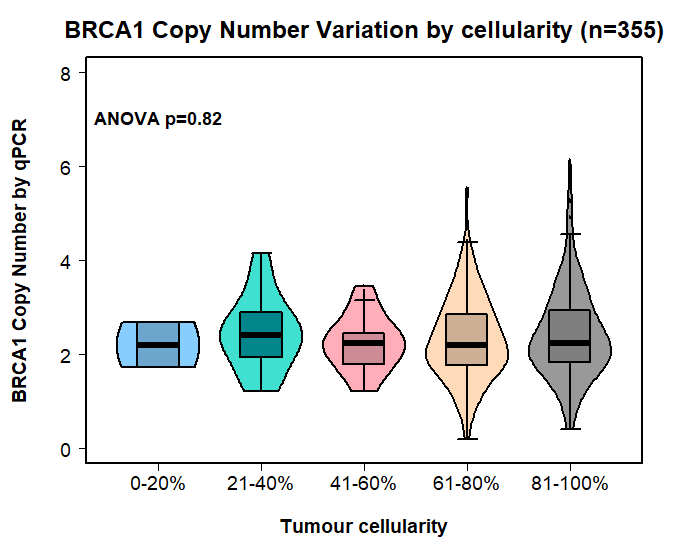

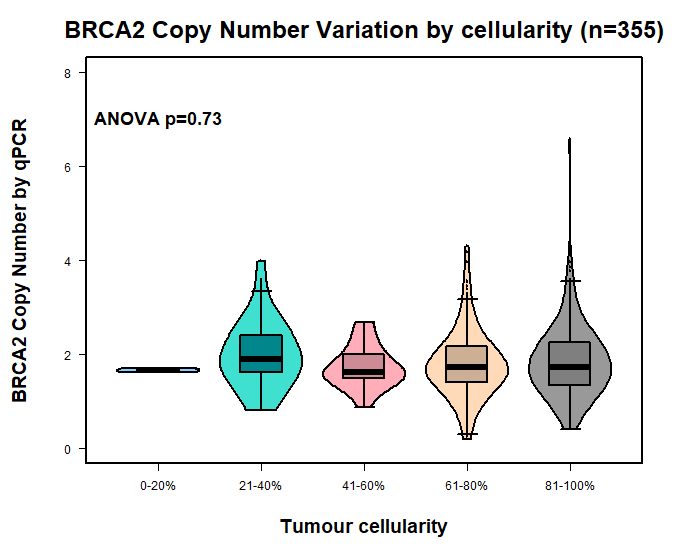


**Supplementary Figure 3**. **(A)** Distribution of tumour cellularity (%) by pathological review of samples of cohort 1. **(B)** Copy number variation of *BRCA1* and *BRCA2* measured by qPCR in samples grouped by their tumour cellularity percentage. Sample size for tumour cellularity subgroups; 0-20% cellularity (n=2, 0.6%), 21-40% cellularity (n=27, 7.6%), 41-60% cellularity (n=18, 5.1%), 61-80% (n=141, 39.8%), 81-100% cellularity (n=168, 47.3%).

**A**


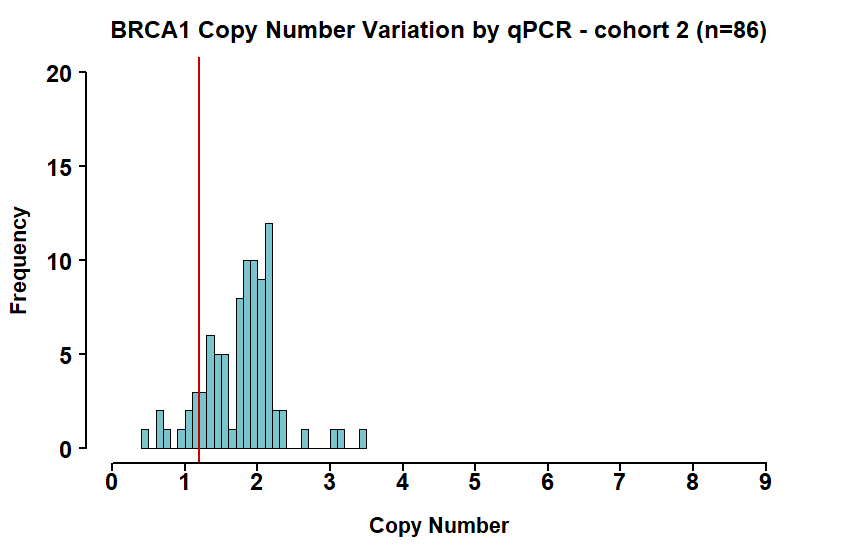

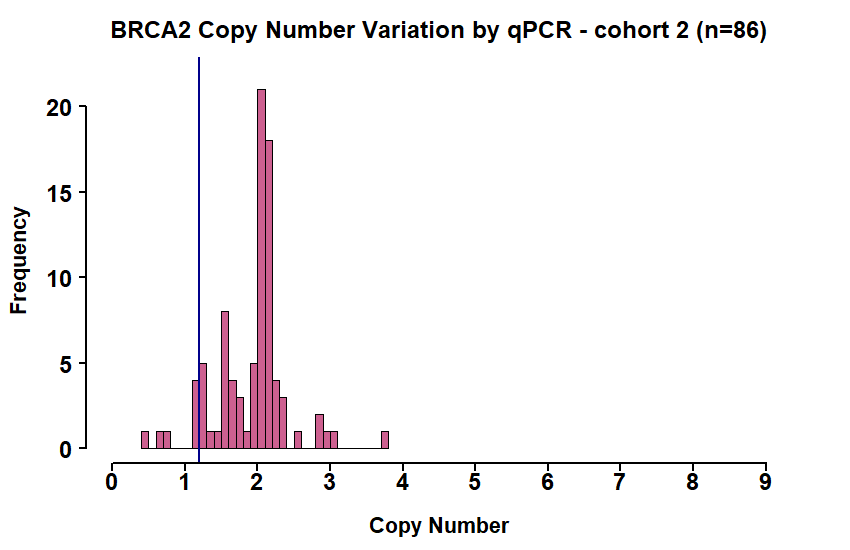


**B**


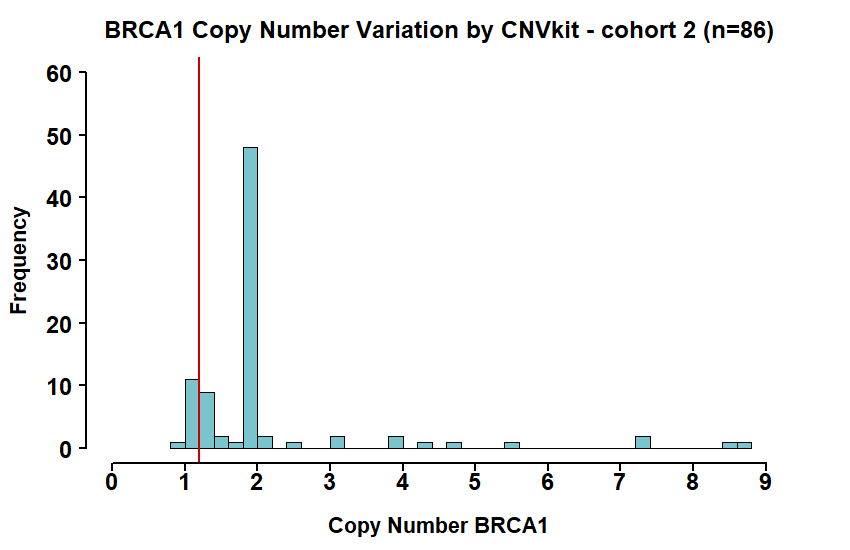

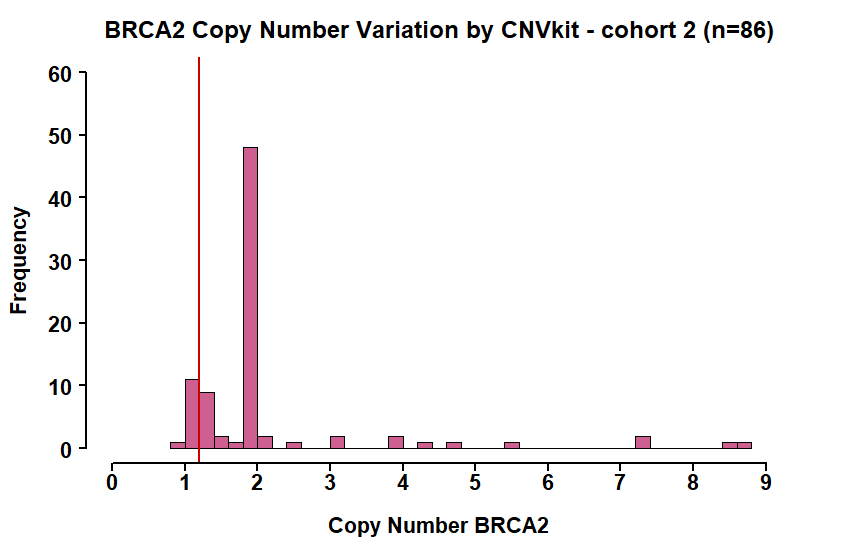


**Supplementary Figure 4**. **(A)** Distribution of *BRCA1* (left) & *BRCA2* (right) absolute copy number by qPCR in cohort 2. The red/blue line indicates the threshold for considering copy number loss (absolute copy number of 1.2). The median copy number for *BRCA1* was 1.87 ± 0.50 and 2.06 ± 0.51 for *BRCA2*. **(B)** Distribution of *BRCA1* (left) & *BRCA2* (right) absolute copy number by CNVkit in cohort 2. The red/blue line indicates the threshold for considering copy number loss (absolute copy number of 1.2). The median *BRCA1* copy number was 2.00 ± 1.49 and median *BRCA2* copy number was also 2.00 ± 1.54 by CNVkit.
